# Early Chronic Carbamazepine-in-Food Administration to MAM/Pilocarpine Rats Does Not Affect Convulsive Motor Seizures

**DOI:** 10.3389/fphar.2020.00181

**Published:** 2020-02-28

**Authors:** Paola Nobili, Alessandro Cattalini, Ugo de Grazia, Cinzia Cagnoli, Marco de Curtis, Giorgio Stefano Battaglia, Francesca Colciaghi

**Affiliations:** ^1^ Clinical and Experimental Epileptology Unit, Fondazione IRCCS Istituto Neurologico Carlo Besta, Milano, Italy; ^2^ Laboratory of Neurological Biochemistry and Neuropharmacology, Fondazione IRCCS Istituto Neurologico Carlo Besta, Milano, Italy

**Keywords:** malformation of cortical development, cortical dysplasia, double-hit model, seizures, drug-resistance, drug-in-food protocol, brain damage

## Abstract

Antiepileptic drug-resistance is a major health problem in patients with cortical dysplasia (CD). Whether drug-resistant epilepsy is associated with progressive brain damage is still debated. We previously generated a rat model of acquired CD, the methylazoxymethanol-pilocarpine (MP) rat, in which the occurrence of status epilepticus and subsequent spontaneous seizures induce progressive brain damage (Nobili et al., 2015). The present study tested the outcome of early-chronic carbamazepine (CBZ) administration on both seizure activity and brain damage in MP rats. We took advantage of the non-invasive CBZ-in-food administration protocol, established by Ali (2012), which proved effective in suppressing generalized convulsive seizures in kainic acid rat model of epilepsy. MP rats were treated immediately after the onset of the first spontaneous seizure with 300 mg/kg/day CBZ formulated in pellets for a two-months-trial. CBZ-treated rats were continuously video-monitored to detect seizure activity and were compared with untreated epileptic MP rats. Despite CBZ serum levels in treated rats were within the suggested therapeutic range for humans, CBZ affected spontaneous convulsive seizures in 2 out of 10 treated rats (responders), whereas the remaining animals (non-responders) did not show any difference when compared to untreated MP rats. Histological analysis revealed cortical thinning paralleled by robust staining of Fluoro-Jade^+^ (FJ^+^) degenerating neurons and diffuse tissue necrosis in CBZ-non-responder *vs* CBZ-responder rats. Data reported here suggest that MP rat model represents suitable experimental setting where to investigate mechanisms of CD-related drug-resistant epilepsy and to verify if modulation of seizures, with appropriate treatment, may reduce seizure-induced brain damage.

## Introduction 

Malformations of cortical development are the results of different pathologic events occurring during the process of cortical ontogenesis (Barkovich et al., 2012) and represent the neuropathologic substrate of a large proportion of drug-resistant epileptic patients. In particular, focal cortical dysplasia (FCD) is the most common brain malformation in patients undergoing epilepsy surgery for the relief of intractable seizures (Fauser et al., 2006; Lerner et al., 2009; Blümcke et al., 2011; Battaglia et al., 2013). Drug-resistance represents a still unsolved major clinical issue since the only determined intervention for pharmaco-resistant epileptic patients is the surgical resection of the epileptic focus, whenever possible. For this reason, the identification of drug-resistance mechanisms and the development of new therapeutic strategies overcoming resistance problem are crucial issues that need to be investigated in order to improve epileptic patients’ outcome.

Prenatal administration of methylazoxymethanol (MAM) in rats at E15 is capable of inducing microcephaly, alteration of apical and basal dendritic morphology, loss of lamination and para-/intra-hippocampal heterotopia in all newborns, therefore MAM rats have been long used to model human brain malformations (Chevassus-au-Louis et al., 1998; Colacitti et al., 1999; Calcagnotto and Baraban, 2005; Battaglia et al., 2017). However, MAM rats suffer from the virtual lack of seizure occurrence and, despite studies exploring and demonstrating hyperexcitability, they rarely develop spontaneous seizures (Harrington et al., 2007; Battaglia et al., 2017). To study the mechanisms of epileptogenicity in the malformed brain, we previously developed a rat model of acquired cortical dysplasia based on prenatal MAM exposition and post-natal pilocarpine treatment (MAM-pilocarpine or MP rat), leading to status epilepticus (SE) and subsequent spontaneous recurrent seizures [SRS; (Colciaghi et al., 2011)]. MP rat model recapitulates both pathological conditions of human CD, i.e., abnormal cortical structure and SRS. We showed that SRS in this experimental model are associated with progressive cellular and molecular brain abnormalities (Colciaghi et al., 2011; Colciaghi et al., 2014). More recently, we showed that cell death after SE, in MP rat model, takes place during the entire epilepsy course, and we reported a temporal and regional specific pattern of brain damage, that is more evident in neocortex and thalamus in the early epilepsy stages (3 days after SRS onset), and becomes predominant in hippocampal CA layers in the later chronic stages (3–6 months of SRS), supporting the hypothesis that the widespread neurodegenerative process, may be related to seizure recurrence (Nobili et al., 2015). The question whether epilepsy is a static or progressive disease has been long-debated and still unsolved (Cole, 2000; Sutula et al., 2003; Finardi et al., 2013; Rossini et al., 2017). In fact, although recurring seizures can potentially contribute to neuronal reorganization and eventually cell death, data from both experimental and human studies are somewhat controversial. At the same time, evidence of progressive neurodegeneration in resistant epilepsy would support early surgical intervention (Haneef et al., 2010). To address this issue, appropriate anti-seizure treatment should be designed to independently evaluate long-term effects of chronic seizures on brain structure/function from those related to SE occurrence (Holmes et al., 1998). The comparison in a given experimental model between SE/no-SRS and SE/SRS animals should demonstrate the effect of seizures in the chronic epileptic phase on the brain.

Therefore, here we tested the outcome of early-chronic carbamazepine (CBZ) administration on both seizure activity and brain damage in MAM-pilocarpine rats, taking advantage of the non-invasive CBZ-in-food administration protocol [300 mg/kg body weight (bwt)/daily] which proved effective in completely suppressing generalized convulsive seizures in non-malformed kainic acid rat model of epilepsy (Ali et al., 2012).

## Materials and Methods

### Ethical Statement

According to the ARRIVE guidelines, procedures were carried out to minimize discomfort and pain to treated rats, in compliance with National (D.L. 116 Suppl 40/1992 and D.L. 26/2014) and International guidelines and laws (2010/63/EU Legislation for the protection of animals used for scientific purposes). The experimental protocols were approved by the Ethics Committee of the Fondazione IRCCS Istituto Neurologico C. Besta and by the Italian Ministry of Health (protocol numbers: BR1/2012 and 961/2016-PR). Rats were housed in groups of two or three per cage under controlled conditions (22 ± 2°C and 12:12 light–dark cycle, lights on at 7 a.m.) until pilocarpine-SE induction. From this moment onwards, they were individually housed in cages and they were kept separated for all the duration of the trial. The animals were given free access to food and water.

### MAM and Pilocarpine Administration

Pregnant Sprague-Dawley rats (n = 4; Charles River, Calco, Italy) received two intraperitoneal (ip) doses of MAM (15 mg/kg maternal body weight, in sterile saline, MRI Global, Kansas City, Missouri, USA) 12 h apart at embryonic day E15, as previously reported (Colacitti et al., 1999). Twenty-eight young adult litters (280–350 g, 2–3 months old) of MAM-treated dams were used for inducing SE with pilocarpine (270 mg/kg ip), as previously described (Colciaghi et al., 2011). Thirty minutes before pilocarpine, rats received N-methylscopolamine (1 mg/kg, ip) to minimize peripheral cholinergic activation (Clifford et al., 1987).

### SE and Seizure Assessment

Pilocarpine treated rats were monitored by two researchers that rated pilocarpine-induced symptoms. Briefly, behavioral seizures were graded as non-convulsive seizures (NCS) and convulsive motor seizures [CMS; tonic-clonic, tonic, and clonic; (Williams et al., 2009)] according to a modified Racine’s scale (Sharma et al., 2018; Colciaghi et al., 2019). SE onset was defined by the time of pilocarpine injection to the occurrence of the first CMS (stage 3–5) followed by continuous seizure activity: once rats reached stage 3–5, the behavioral activity fluctuated between NCS (stage 1–2) to CMS (stage 3–5). Rats that experienced either one isolated stage 3–5 seizure or stage 1–2 NCS only and that did not develop continuous seizure activity were excluded from the study (n = 6 rats, 22% of pilocarpine-treated rats). Diazepam (10 mg/kg ip) was administered 90 min after SE onset to alleviate seizures and decrease mortality rate. Death occurring during SE (n = 6 rats, 27% of MP rats) could not be anticipated on the basis of external monitoring. MAM-pilocarpine treated rats were continuously video-recorded using an infrared camera system (24h/day for 2 months) to detect the onset of spontaneous seizures and to quantify SRS occurrence. SRS were graded as follows: stage 0 through 5, as outlined by Racine (1972); stage 6, cluster of multiple stage 5 seizures; stage 7, violent jumping and running; stage 8, stage 7 plus tonic hindlimb extension and tail rigidity (Pinel and Rovner, 1978a; Pinel and Rovner, 1978b). Two researchers blinded to rats’ treatment observed the videos in a fast-forward mode. In case of any seizure-like activity, the videos were stopped, reversed and watched at normal speed. Since we could just rely on video-monitoring, we chose to restrict seizure quantification to generalized convulsive motor seizures (stages 4-7). Stage 8 seizures were never observed during video-monitoring of chronic epileptic rats. Total amount of generalized convulsive motor seizures were quantified for 50 days of video-recording/each rat. However, during video-monitoring analysis, any event suggestive of focal seizure (Racine stage 1-3, such as orofacial automatisms, head nodding, anterior limb clonus with lordotic posture) together with wild running/jumping behavioral not eventually evolving in CMS, were noted for each rat.

### Carbamazepine-in-Food Treatment

Randomly chosen epileptic MP rats surviving SE were treated immediately after the onset of the first spontaneous seizure with a daily dose of 300 mg/kg (bwt) CBZ formulated in food pellets for two-months (n = 10, hereafter referred to as MP-CBZ rats), according with protocol previously established by Ali and colleagues (Ali et al., 2012). Since the amount of food-intake for the maintenance of body weight of an adult rat is about 60 g/kg bwt/day, CBZ was formulated in food pellets as 5 mg CBZ per 1 g pellet (Mucedola Srl, Milano, Italy). CBZ-containing food (60 g pellet/kg bwt) was supplied in a single feeding each day at 10 a.m. for a total of 60 days (60 administrations). CBZ-treated rats were weighed every 3–5 days to determine any possible effect of CBZ-containing pellet on body weights. Further, the residual pellet possibly left was weighed every day in order to calculate the exact amount of pellet and CBZ dose taken by the individual rat/each day. In order to estimate the bioavailability of the drug, the CBZ serum level was estimated in each individual rat the last day of treatment using the Carbamazepine assay on the ARCHITECT c Systems™ (iCARB-Enzyme immunoassay, Abbott Laboratories), according to product insert and routinely used to monitor CBZ serum level in patients.

Remaining MP rats (n = 6) were used as proper epileptic untreated rat group and received normal pellet (hereafter referred as MP-untreated rats). Additional n = 4 age-matched naïve MAM rats, neither experiencing SE nor SRS, were used as untreated non-epileptic controls (MAM-CTR). At the end of the two-months-trial, all rats were sacrificed for histological/morphometric analysis (see below).

### Morphologic Analysis

Morphologic analysis was performed as previously described (Colciaghi et al., 2019). Briefly, animals were killed with overdose of chloral hydrate and then perfused with 4% paraformaldehyde in 0.1 M phosphate buffered saline at pH 7.2. Brains were removed from the skull, post-fixed overnight, and cut with vibratome (Leica Biosystem, Wetzlar, Germany) into 40 to 50 µm thick coronal sections that were collected in serial order. As morphologic/neuroprotective read-outs of chronic CBZ-therapy, we evaluated the cortical thickness in thionine stained sections (see data analysis section, below) and the Fluoro-Jade (FJ; Histo-Chem Inc., Jefferson, AR, USA) labeling in cortex and hippocampus (Nobili et al., 2015). One series of coronal sections (1 out of 7 sections) was counterstained with 0.1% thionine and at least 6 sections per rat (from -0.3 to -5.8 mm from bregma) were processed for FJ analysis as previously reported (Nobili et al., 2015).

### Data Analysis and Statistical Evaluation

Latency to SE onset and SE behavioral evolution were analyzed in MP-untreated *vs* MP-CBZ rat group and statistically compared by means respectively of Mann-Whitney non parametric U-test and Student’s t-test. For CMS (stage 4–7) quantification, since MP rats exhibited spontaneous seizure activity with a non-normal distribution (regardless of control or CBZ treatment), a Mann-Whitney U-test was used to compare the total number of seizures between the two experimental groups.

Cortical thickness was measured in 3 serial thionine-stained coronal sections from i) the rostral cortex (anterior commissure, at ~ −0.3/−0.8 mm from bregma), ii) the somatosensory frontoparietal cortex (−2.8/−3.8 mm from bregma), iii) the temporal or posterior cortex (−4.8/−5.8 mm from bregma) (Paxinos and Watson, 1982). Selected sections were digitized by means of Aperio CS2 slide scanner (Leica Biosystems Nussloch GmbH) at 20x magnification and cortical thickness was measured in each section at 0° (1 mm lateral to the midline), 45° and 90° from the midline, as previously described (Colciaghi et al., 2011; Colciaghi et al., 2014) and indicated in **Figure 2A**. The three measures per section were averaged to a single value and the obtained measures from the 3 serial sections from each area were averaged again to a single value to obtain the mean cortical thickness of rostral, sensorimotor and posterior cortex for each rat. N = 4 MAM-CTR, n = 8 MP-CBZ and n = 6 MP-untreated rats were analyzed. Differences among groups were statistically analyzed for each neocortical area by means of one-way analysis of variance (ANOVA) followed by Tukey HSD as post-hoc comparison test.

All measurements were performed independently by two operators blind the to the animal treatment; data were expressed as mean ± SD (or SEM when indicated) and differences were considered significant with p < 0.05. The sample size of rats necessary to detect a difference of 15% with a power of 80% and alpha 0.05 (Machin et al., 1997) between MP untreated and MP-CBZ groups was estimated using variance values obtained in previous similar cortical thickness determinations (Colciaghi et al., 2014; Nobili et al., 2015) and data of video-monitoring seizure analysis in chronic epileptic pilocarpine/kainic rats treated with CBZ reported by other groups (Chakir et al., 2006; Polli et al., 2014).

## Results

### Seizure Assessment and Selection of CBZ-Responders and Non-Responders

**Figure 1:**
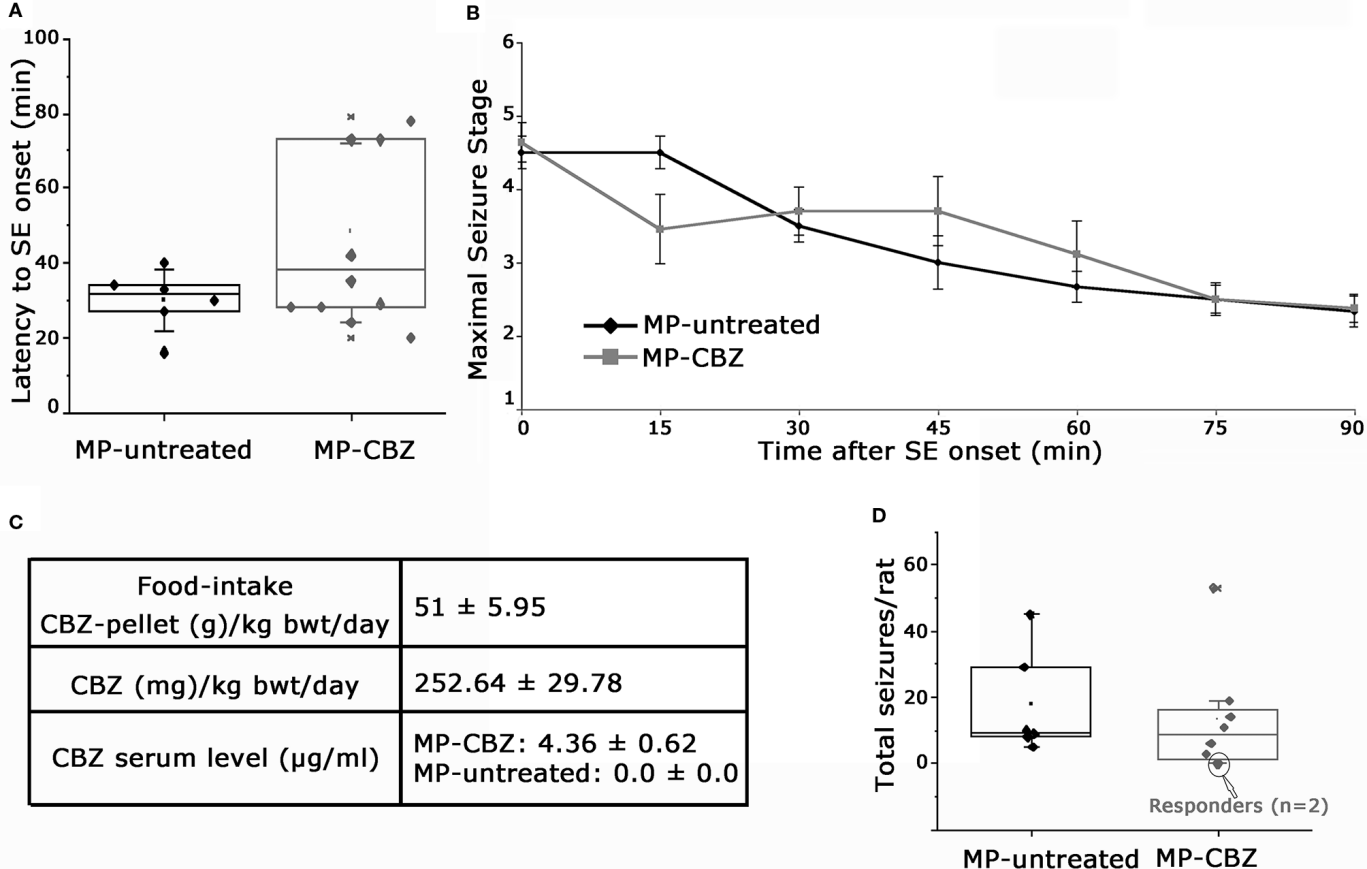
Behavioral assessment of status epilepticus and SRS and data of CBZ-in-food trial. **(A)** Box plot representation of latency (min) to onset of pilocarpine-induced SE in MP-untreated and MP-CBZ treated rats. No significant difference was observed between MP-CBZ and MP-untreated rats as assessed by means of Mann-Whitney U-test (respectively 30 ± 8.25 min *vs* 46 ± 23.77 min, p = 0.242). **(B)** Behavioral assessment of convulsive-motor seizure (stage 3-5) throughout status epilepticus, rated every 15 min over a 90 min observation period. No differences were observed between MP-untreated and MP-CBZ rat group at each time point considered as assessed by student t-test (SE onset, 0 min: p = 0.71; 15 min: p = 0.125; 30 min: p = 0.45; 45 min: p = 0.276; 60 min: p = 0.435; 75 min: p = 1; 90 min: p = 0.884; n = 6 rats in MP-untreated group; n = 10 rats in MP-CBZ group). Data are expressed as mean ± SD. **(C)** CBZ administration started after the first spontaneous seizure and the drug was administered in food every 24 h (at 10 a.m.) for 2 months. The food intake corresponded to a normal caloric diet. Average CBZ dose per day was 252.64 ± 29.78 mg/kg/day (mean ± SD) and serum CBZ levels were within the range suggested for humans (4–12 μg/ml). **(D)** Box plot showing the total number of stage 4-7 CMS/per rat during the trial. No difference was observed between the two groups as assessed by means of Mann-Whitney U-test (mean SRS number ± SEM: 17.15 ± 6.48 and 13.23 ± 6.16, respectively for MP-untreated and MP-CBZ rat group, p = 0.560). Encircled dots in panel D represent 2 out of 8 MP-CBZ rats (MP-CBZ/responders) in which CBZ treatment totally affected convulsive stage 4-7 seizures. In A and D, the box ranges indicate the 25th and 75th percentile and whiskers represent SD values.

MP rats were randomly assigned to untreated- or CBZ-experimental group: a retrospective analysis of SE evolution of single rats was conducted to verify possible difference of SE severity between the two groups (**Figures 1A, B**). SE onset time ranged between 16–70 min after pilocarpine injection. We did not observed differences in regards to latency to SE onset between MP-untreated and MP-CBZ rat groups (respectively 30 ± 8.25 min *vs* 46 ± 23.77 min, p = 0.242 **Figure 1A**). All rats were scored every 15 min after the onset of the first CMS (stage 3–5, *Time 0* in **Figure 1B**): no differences in seizure development and intensity according to the behavioral scoring of CMS were observed between the CBZ-treated and untreated-rats (**Figure 1B**). The estimated SRS onset was not significantly different between the two groups: 6.43 ± 1.50 and 11.15 ± 4.30 (day after SE ± SD), respectively for MP-CBZ and MP-untreated rats. The day after SRS onset, rats assigned to MP-CBZ experimental group (n = 10) entered the two-months CBZ trial. Two out of 10 MP-CBZ treated rats died due to stage 7 seizures, respectively 3 and 5 days after SRS onset. Data obtained in the CBZ trial (n = 8 rats) are reported in **Figure 1C** and can be summarized as follows: all rats ate the CBZ-containing food throughout the 24 h day irregularly but continuously, with feeding episodes concentrated in the first 2 h after providing the CBZ pellet (10–12 a.m.) and in the first 2–3 h of the dark period (7–10 p.m.). The total amount of food consumed per day corresponded to a normal caloric diet (food-intake, **Figure 1C**), and no difference was detected in the progressive increase of body weight among the treated rats during CBZ treatment (data not shown). Mean CBZ dose taken/day per rat was 252.64 ± 29.78 mg/kg/day (mean ± SD) while serum CBZ level in treated rats was 4.36 ± 0.60 μg/ml, within the suggested therapeutic range for humans [4–12 μg/ml; (Patsalos et al, 2018)].

The quantification of stage 4–7 convulsive motor seizures (Pinel and Rovner, 1978a; Pinel and Rovner, 1978b) during 2 months of video recording was not significantly different between MP-untreated and MP-CBZ rats (**Figure 1D**). SRSs occurred within clusters followed by seizure-free period for both groups; no differences were found between treated *vs* untreated rats [mean SRS cluster interval (days) ± SD: MP-untreated: 1.84 ± 1.13; MP-CBZ: 2.35 ± 1.80; n.s. p = 0.56; mean inter-cluster interval (days) ± SD: MP-untreated: 15.30 ± 5.8; MP-CBZ: 10.11 ± 5.96; n.s. p = 0.429]. However, CBZ treatment affected stage 4–7 CMS in 2 out of 8 MP-CBZ rats (encircled dots in panel D), hereafter referred to as MP-CBZ/responders. SRS onset time of the two MP-CBZ/responders was respectively 5 and 6 days post-SE.

It’s noteworthy that all rats, regardless of the treatment, daily exhibited events suggestive of focal seizures, such as orofacial automatisms, head nodding and anterior limb clonus with lordotic posture, together with periods of spontaneous and intense wild running or jumping-like behavior without generalized convulsion mainly concentrated in the dark period. In the absence of electroencephalography (EEG) monitoring these events were not included in seizures’ quantification.

### Morphological Assessment of SRS and CBZ Treatment Effect on the Epileptic Brain

**Figure 2:**
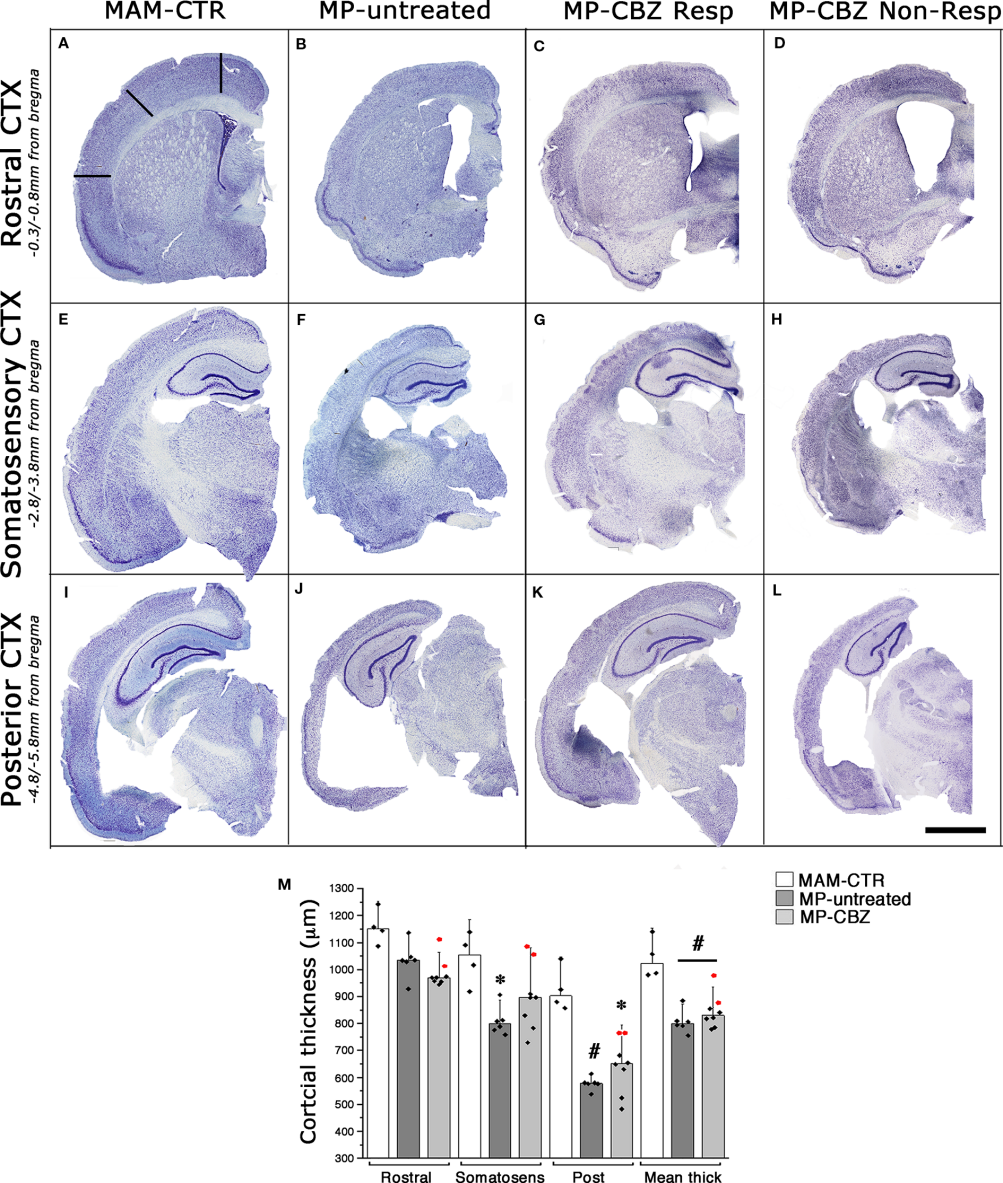
Effects of seizures and CBZ treatment on neocortical atrophy. **(A–L)** Low-power thionine-stained coronal sections from rostral **(A–D)**, somatosensory **(E–H)** and posterior **(I–L)** cortical areas in representative non-epileptic naïve MAM (MAM-CTR; **A, E, I)**, epileptic MP-untreated **(B, F, J)**, MP-CBZ/responder **(C, G, K)** and MP-CBZ/non responder rats **(D, H, L)**. Scale-bar: 2mm **(M)** Bar-chart with dot-plots of single measurements of cortical thickness analyzed in different cortical region in MAM-CTR (n = 4), MP-untreated (n = 6) and MP-CBZ rat (n = 8) groups. Cortical thickness was significantly affected in the posterior cortex (Post) in both MP-untreated (^#^p < 0.01) and MP-CBZ (* p < 0.05) *vs* MAM-CTR rats. Mean cortical thickness (Mean thick) was significantly decreased in both MP-untreated and MP-CBZ rats when compared to MAM-CTR rats (^#^p < 0.01). A trend to decrease, although not significant, was observed in the rostral and somatosensory cortical areas in MP-CBZ *vs* MAM-CTR. Differences never emerged by comparing MP-CBZ with MP-untreated rats. Red-dots indicate the single cortical measurements of the 2 rats in which CBZ treatment totally affected convulsive stage 4–7 seizures. Data were expressed as mean ± SD.

We previously showed that the occurrence of SE plus SRS affects the cytoarchitecture of the chronic epileptic MAM/pilocarpine rat brain, significantly reducing both cortical thickness and hippocampal volume after 3 months of SRS (Colciaghi et al., 2011; Colciaghi et al., 2014). We therefore evaluated the effect of recurrent seizures on brain morphology by comparing the cortical thickness of non-epileptic naïve MAM (MAM-CTR), MP-untreated and MP-CBZ rats (**Figure 2**). Representative serial coronal thionine stained sections from each experimental group are reported in **Figures 2A-L**. The mean cortical thickness was significantly reduced in both MP-untreated and MP-CBZ rats when compared to non-epileptic MAM-CTR rats, mainly affecting the posterior temporal and entorhinal cortex (**Figure 2M**). In contrast, no significant difference was obtained between MP-CBZ and MP-untreated rats.

**Figure 3:**
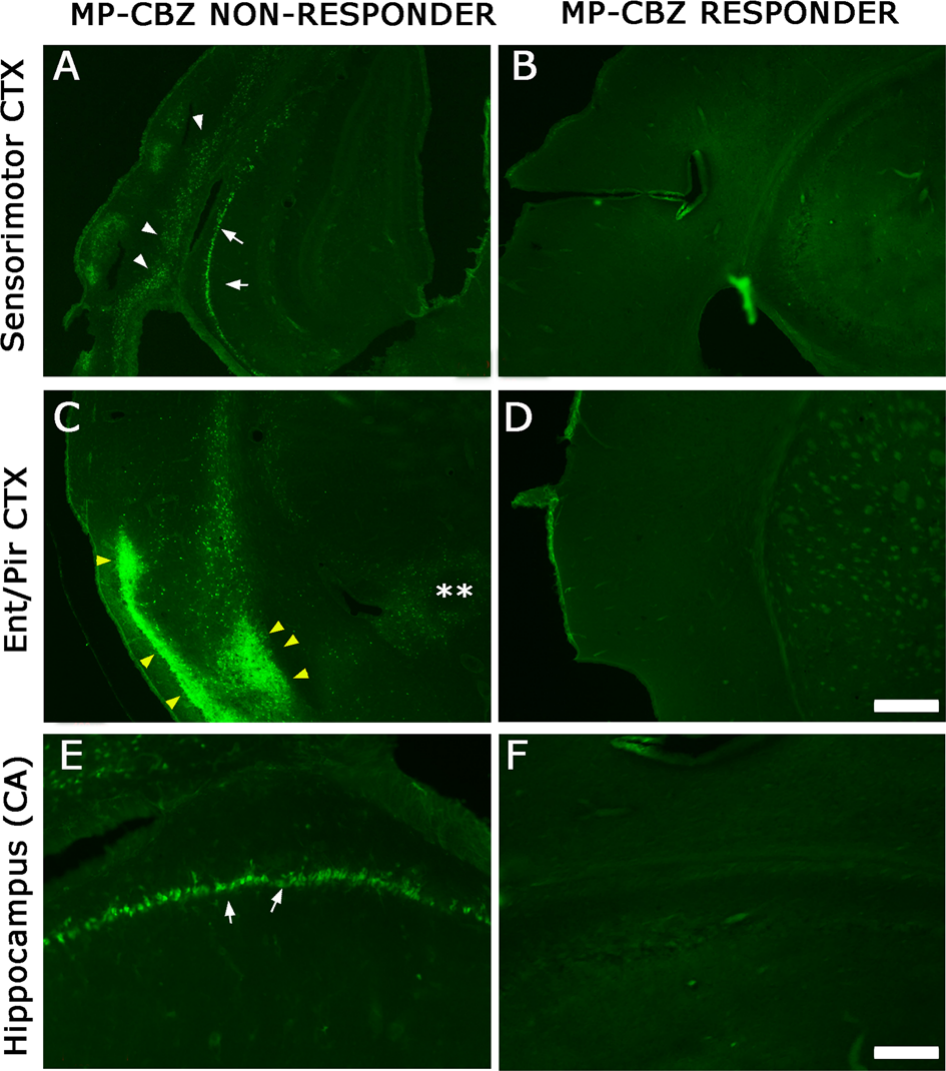
FJ labeling in neocortical and hippocampal areas in MP-CBZ/non-responders and MP-CBZ/responders. FJ staining of representative MP-CBZ/non-responder **(A**, **C**, **E)** and MP-CBZ/responder rat **(B**, **D**, **F)** in the frontoparietal sensorimotor **(A, B)**, and entorhinal **(C, D)** cortical areas, and in the hippocampus **(E, F)**. Note the numerous FJ^+^ neurons in all neocortical areas in MP-CBZ/non-responder when compared to MP-CBZ/responder rats. FJ^+^ degenerating neurons were found in deep cortical layers of sensorimotor cortex (white arrowheads, **(A)** of MP-CBZ/non-responder rats, paralleled by diffused necrotic areas at the entorhinal/piriform cortical area (yellow arrowheads in **(C)**. At the hippocampal level many FJ positive neurons were detected in CA1/2 regions of MP-CBZ/non-responder rats (arrows in **A**, **E**). By contrary, FJ^+^ degenerating neurons were never found in MP-CBZ/responder rats in any region considered (2 out of 8; **B**, **D**, **F**). Scale-bars: 300 μm in A-D; 100 μm in E-F.

To evaluate the localization and extent of degenerating neurons, FJ labeling was performed in MP-CBZ/responder and non-responder rats (**Figure 3**). We did not detect any FJ^+^ neuron in CBZ-responders (**Figures 3B, D, F**), while CBZ/non-responder rats (**Figures 3A, C, E**) exhibited a FJ^+^ pattern very similar to that previously demonstrated in chronic epileptic MP rats (Nobili et al., 2015). In particular, evident labeling was obtained in deep cortical layer (arrowheads, **Figure 3A**) paralleled by intense staining of hippocampal CA pyramidal neurons (arrows **Figures 3A, E**) and by the presence of diffuse necrosis in entorhinal cortex (yellow arrowheads, **Figure 3C**).

## Discussion

The present study extends the characterization of MAM/pilocarpine rat model of epileptogenic cortical dysplasia, previously established in our lab (Colciaghi et al., 2011; Colciaghi et al., 2014; Nobili et al., 2015), revealing no effect of early chronic administration of CBZ 300 mg/kg/day (2-months trial) on generalized convulsive motor seizures and on brain damage in most treated rats (8 out of 10) when compared to untreated epileptic MP rats.

### Limitations of the Study

The findings of this study have to be seen in light of some limitations. The primary concerns the use of video-monitoring for spontaneous seizure assessment. In fact, since we could not perform video-EEG monitoring to indisputably identify focal seizures (Racine 1–3 seizure stage) that could be confused with non-epileptic movements related to normal rat activity, the anti-seizure efficacy of CBZ was restricted to the quantification of generalized CMS (stage 4–7). Similarly, activities suggestive of a seizure, such as wild-running behavior without falling, lordosis, tail erection were included in our quantification only when evolving into convulsive tonic-clonic seizures. The exclusion of <3 Racine seizures (and the consequent under-sampling of seizures) is likely the main reason for the relative low number of reported seizures in our limited setting (without video-EEG). The analysis of CMS by video-monitoring was the most reliable readout available to us. Thus, we believe that under-sampling in our experimental conditions was a necessity. However, taking into account that CBZ-in-food-protocol (300 mg/kg/day) was totally effective in suppressing any event suggestive of a seizure in epileptic kainic acid rat model (Ali et al., 2012), that CMS frequency obtained in untreated-MP rat model is similar to what reported by other groups in long-term (50-60 days) video-monitoring of chronic epileptic pilocarpine and kainic rats (Capella and Lemos, 2002; Chakir et al., 2006; Polli et al., 2014), and that early-chronic treatment with CBZ (120 mg/kg/day) in naïve pilocarpine rats was effective in suppressing Racine stage 4-5 CMS (Chakir et al., 2006), we believe that, although preliminary, the present data could offer an experimental paradigm to investigate mechanisms of resistance to specific AED.

### MAM/Pilocarpine Rat, Model of Pharmaco-Resistant CD

The matter of the drug-resistant epilepsy associated with cortical dysplasia has been broadly addressed in the last years in experimental models, including MAM rats, by assessing the efficacy of conventional AEDs (Smyth et al., 2002; Jellett et al., 2015; Battaglia et al., 2017). In general, most of the studies showed a basic medically refractoriness of MAM rats to these conventional AEDs but virtually nothing is known about chronic AED treatment on seizure expression and seizure-related brain damage in epileptic malformed rats. *In-vitro* experiments showed that epileptiform activity induced by potassium channel blocker 4-Aminopyridine (4-AP) in hippocampal slices from MAM rats was relatively refractory to phenobarbital, CBZ, valproate (VPA), ethosuximide (ESM) and lamotrigine. Accordingly, *in vivo* experiments demonstrated that VPA was not effective in prolonging latency to SE in MAM animals after acute administration of kainic acid, in contrast to what observed in control animals (Smyth et al., 2002) or when seizures were induced by flurothyl inhalation (Jellett et al., 2015). In 2004, Serbanescu and colleagues, by combining prenatal MAM exposure with postnatal treatment with cholesterol biosynthesis inhibitor AY-9944, generated the two-hit MAM-AY rat model of chronic atypical absence seizures (Serbanescu et al., 2004). ECoG analysis of slow spike-and-wave discharge (SWD) duration did not show any difference between naïve-AY and MAM-AY rats but revealed medical refractoriness of MAM-AY rats to conventional anti-absence drugs (namely ESM and VPA). However, the analysis was restricted to a single drug administration and SWD quantification was limited to 1h recording period.

As CD patients are characterized by pharmaco-resistant epilepsy, the most valid experimental model, where screening long-term efficacy of AEDs, should rely on chronic and spontaneous seizure activity associated with brain malformation. In our opinion, this is a central issue, never addressed so far. In fact, as properly suggested by Potschka (2012), whether disease-associated alterations occur as consequences of repeated seizures, that—in turn—could contribute to therapeutic failure, should be verified in chronic model with SRS and not merely in acute seizure model, e.g., as latency to seizure onset.

To the best of our knowledge, this is the first study testing the outcome of early-chronic AED administration on both seizure activity and brain damage in CD model. As previously reported by Dudek’s group, the once-per-day non invasive CBZ-in-food administration protocol used in the present study offered the evident advantage of minimizing the handling of epileptic rats (Grabenstatter et al., 2007; Ali et al., 2012), thus avoiding the stress and pain unavoidably associated with ip injections, which in turn could induce seizures (Jöels, 2009) thus resulting in a very practicable approach for preclinical drug screening. As pilot, the present study does not claim to get deep into the possible mechanisms of AED-resistance associated with cortical dysplasia, rather it was originally established to verify whether and to what extent SRS could be controlled in MAM/pilocarpine rats, and to dissect the eventual effect of SE from that of SRS on brain damage. Additional trials, on larger cohort of animals treated with different AEDs, will be necessary to address the last issue. In this regard, preliminary unpublished data obtained from few MP rats (n = 4) from our lab show that spontaneous seizure activity of MP-CBZ non-responder rats was not affected either when rats entered in a subsequent trial with clobazam (CLB)-in food (40 mg/kg/day, for 4 weeks). Taken together these data indicate that MAM/pilocarpine rat can be considered a model of medically-intractable seizure or, at least, very poor response to CBZ.

The dysfunction of blood brain barrier (BBB) and the transporter hypothesis have been proposed as possible contributing factors of such a drug resistance in MAM rats. In fact, an intrinsic BBB leakage is a typical feature of the heterotopic regions of MAM rat brain while no changes were observed in tissue with normal cytoarchitecture (Marchi et al., 2006). Further, the occurrence of pilocarpine-induced SE in MAM rats worsens the BBB damage and induces the up-regulation of the multidrug resistance P-glycoprotein 1 (P-gp) in the perivascular astrocytes (Marchi et al., 2006), similarly to what described in human brain specimens of FCD patients (Sisodiya et al., 1999; Sisodiya et al., 2001; Sisodiya et al., 2002). Additional experiments will be necessary to verify whether dynamic changes in BBB function may determine the pharmacological responses of the epileptic malformed brain to AEDs and whether the BBB is preserved in AED-responder rats.

### Brain Damage in Epilepsy Models: Related to SE Only?

The question whether repeated seizures might be associated with progressive alterations of the brain has been long debated and as yet unresolved. In the current opinion, that a prolonged seizure damages the brain is widely accepted (Sloviter et al., 2006; Sloviter et al., 2007; Noè et al., 2019; Zhang et al., 2012), whereas the hypothesis that seizure recurrence is associated with cumulative brain damage is much more debated, even if supported by recent MRI studies and meta-analysis of MRI morphometry studies in pharmaco-resistant temporal lobe epilepsy patients [TLE; (Bernhardt et al., 2009; Caciagli et al., 2017; Galovic et al., 2019)]. The analysis of convulsive epilepsy models in which seizures are prevented by appropriate treatments would provide a more reliable evaluation of the effect of SE on the brain cellular and molecular features.

MAM/pilocarpine rats represent a convulsive model of chronic epilepsy, since the development of SE is the necessary pre-requisite to determine the later onset of SRS. In this model, therefore, as in most post-SE epilepsy models currently in use, it is difficult to dissect the pathologic effect of SE on the cellular and molecular features of the malformed brain from that of subsequent SRS.

The comparison of brain damage, through already developed morphologic and functional read-outs (Colciaghi et al., 2011; Nobili et al., 2015) revealed that CBZ-responder rats, that experienced frank SE but not stage 4–7 CMS, were similar to non-epileptic MAM-CTR rats. Indeed, FJ^+^ degenerating neurons were never detected in MP-CBZ/responder rats in any region considered. Conversely, the degree of brain damage in CBZ-non responders, that fully developed spontaneous convulsive seizures after SE, was similar to MP-untreated rats but significantly different from MAM-CTR rats. The small number of MP-CBZ responder rats (n = 2) did not allow a statistical comparison of seizure-induced brain damage *vs* other groups. We are aware that the lack of evident brain damage observed in the 2 CBZ-rats could be interpreted as a fortuitous event. However, we believe it’s worth pointing out since it may also suggest that even infrequent spontaneous CMS, and not merely the occurrence of SE, could eventually contribute to brain damage.

## Data Availability Statement

All datasets generated for this study are included in the article/supplementary material.

## Ethics Statement

Procedures were carried out to minimize discomfort and pain to treated rats in compliance with National (D.L. 116 Suppl 40/1992 and D.L. 26/2014) and International guidelines and laws (2010/63/EU Legislation for the protection of animals used for scientific purposes). The experimental protocols were approved by the Ethics Committee of the Fondazione IRCCS Istituto Neurologico Carlo Besta and by the Italian Ministry of Health (protocol numbers: BR1/2012 and 961/2016-PR).

## Author Contributions

GB and FC designed and supervised the study research. FC and MC wrote the paper. PN, CC and FC performed animal treatment. FC, PN, and AC analyzed video-recordings. PN and AC performed/analyzed morphological experiment. FC performed stat analysis. UG analyzed drug plasma level.

## Funding

This work was supported by research grant AICEFIRE2012 and AICE-FIRE2016 (Italian Association Against Epilepsy and Italian Foundation for Epilepsy Research) respectively to GB and FC.

## Conflict of Interest

The authors declare that the research was conducted in the absence of any commercial or financial relationships that could be construed as a potential conflict of interest.

## References

[B1] Ali A. Dua Y. Constance J. E. Franklin M. Dudek F. E. (2012). A once-per-day, drug in-food protocol for prolonged administration of antiepileptic drugs in animal models. Epilepsia 53, 199–206. 10.1111/j.1528-1167.2011.03314.x 22092257

[B2] Barkovich A. J. Guerrini R. Kuzniecky R. I. Jackson G. D. Dobyns W. B. (2012). A developmental and genetic classification for malformations of cortical development: update 2012. [Review] Brain 135, 1348–1369. 10.1093/brain/aws019 22427329 PMC3338922

[B3] Battaglia G. Colciaghi F. Finardi A. Nobili P. (2013). Intrinsic epileptogenicity of dysplastic cortex: converging data from experimental models and human patients. Epilepsia 54 Suppl 6, 33–36. 10.1111/epi.12272 24001068

[B4] Battaglia G. Finardi A. Nobili P. Colciaghi F. (2017). Dysplasia: MAM, model of developmental epilepsy. Models of Seizures and Epilepsy 2nd Ed. Eds. Pitkänen A. Buckmaster P. S. Galanopoulou A. S. Moshé S. L. (Academic Press), 861–876. 10.1016/B978-0-12-804066-9.00058-4

[B5] Bernhardt B. C. Worsley K. J. Kim H. Evans A. C. Bernasconi A. Bernasconi N. (2009). Longitudinal and cross-sectional analysis of atrophy in pharmacoresistant temporal lobe epilepsy. Neurology 72, 1747–1754. 10.1212/01.wnl.0000345969.57574.f5 19246420 PMC2827310

[B6] Blümcke I. Thom M. Aronica E. Armstrong D. D. Vinters H. V. Palmini A. (2011). The clinicopathologic spectrum of focal cortical dysplasias: a consensus classification proposed by an *ad hoc* Task Force of the ILAE Diagnostic Methods Commission. Epilepsia 52, 158–174. 10.1111/j.1528-1167.2010.02777.x 21219302 PMC3058866

[B7] Caciagli L. Xiao F. Wandschneider B. Koepp M. J. (2017). Imaging biomarkers of anti-epileptic drug action: insights from magnetic resonance imaging. Curr. Pharm. Des. 23 (37), 5727–5739. 10.2174/1381612823666170809113636 28799517

[B8] Calcagnotto M. E. Baraban S. C. (2005). Prolonged NMDA-mediated responses altered ifenprodil sensitivity, and epileptiform-like events in the malformed hippocampus of methylazoxymethanol exposed rats. J. Neurophysiol. 94, 153–162. 10.1152/jn.01155.2004 15772235

[B9] Capella H. M. Lemos T. (2002). Effect on epileptogenesis of carbamazepine treatment during the silent period of the pilocarpine model of epilepsy. Epilepsia. 43 (Suppl.5), 110–111. 10.1046/j.1528-1157.43.s.5.9.x 12121304

[B10] Chakir A. Fabene P. F. Ouazzani R. Bentivoglio M. (2006). Drug resistance and hippocampal damage after delayed treatment of pilocarpine-induced epilepsy in the rat. Brain Res. Bull. 71, 127–138. 10.1016/j.brainresbull.2006.08.009 17113938

[B11] Chevassus-au-Louis N. Ben-Ari Y. Vergnes M. (1998). Decreased seizure threshold and more rapid rate of kindling in rats with cortical malformation induced by prenatal treatment with methylazoxymethanol. Brain Res. 812, 252–255. 10.1016/s0006-8993(98)00932-9 9813354

[B12] Clifford D. B. Olney J. W. Maniotis A. Collins R. C. Zorumski C. F. (1987). The functional anatomy and pathology of lithium-pilocarpine and high-dose pilocarpine seizures. Neuroscience. 23 (3), 953–968. 10.1016/0306-4522(87)90171-0 3437996

[B13] Colacitti C. Sancini G. De Biasi S. Franceschetti S. Caputi A. Frassoni C. (1999). Prenatal Methylazoxymethanol treatment in rats produces brain abnormalities with morphological similarities to human developmental brain dysgeneses. J. Neuropathol. Exp. Neurol. 58, 92–106. 10.1097/00005072-199901000-00010 10068317

[B14] Colciaghi F. Finardi A. Frasca A. Balosso S. Nobili P. Carriero G. (2011). Status epilepticus-induced pathologic plasticity in a rat model of focal cortical dysplasia. Brain 134, 2828–2843. 10.1093/brain/awr045 21482549

[B15] Colciaghi F. Finardi A. Nobili P. Locatelli D. Spigolon G. Battaglia G. S. (2014). Progressive brain damage, synaptic reorganization and NMDA activation in a model of epileptogenic cortical dysplasia. PLoS One 9, e89898. 10.1371/journal.pone.0089898 24587109 PMC3937400

[B16] Colciaghi F. Nobili P. Cipelletti B. Cagnoli C. Zambon S. Locatelli D. (2019). Targeting PSD95-nNOS interaction by Tat-N-dimer peptide during status epilepticus is neuroprotective in MAM-pilocarpine rat model. Neuropharmacology 153, 82–97. 10.1016/j.neuropharm.2019.04.028 31047919

[B17] Cole A. J. (2000). Is epilepsy a progressive disease? The neurobiological consequences of epilepsy. Epilepsia 41 Suppl 2, S13–S22. 10.1111/j.1528-1157.2000.tb01520.x 10885736

[B18] Fauser S. Huppertz H. J. Bast T. Strobl K. Pantazis G. Altenmueller D. M. (2006). Clinical characteristics in focal cortical dysplasia: a retrospective evaluation in a series of 120 patients. Brain 129, 1907–1916. 10.1093/brain/awl133 16714316

[B19] Finardi A. Colciaghi F. Castana L. Locatelli D. Marras C. E. Nobili P. (2013). Long-duration epilepsy affects cell morphology and glutamatergic synapses in type IIB focal cortical. Acta Neuropathol. 126, 219–235. 10.1007/s00401-013-1143-4 23793416

[B20] Galovic M. van Dooren V. Q. H. Postma T. Vos S. B. Caciagli L. Borzì G. (2019). Progressive cortical thinning in patients with focal epilepsy. JAMA Neurol 76, 1230–1239. 10.1001/jamaneurol.2019.1708 PMC6604082 31260004

[B21] Grabenstatter H. L. Clark S. Dudek F. E. (2007). Anticonvulsant effects of carbamazepine on spontaneous seizures in rats with kainite-induced epilepsy: comparison of intraperitoneal injections with drug-in-food protocols. Epilepsia 48, 2287–2295. 10.1111/j.1528-1167.2007.01263.x 17711461

[B22] Haneef Z. Stern J. Dewar S. Engel J. Jr. (2010). Referral pattern for epilepsy surgery after evidence-based recommendations: a retrospective study. Neurology 75 (8), 699–704. 10.1212/WNL.0b013e3181eee457 20733145 PMC2931651

[B23] Harrington E. P. Möddel G. Najm I. M. Baraban S. C. (2007). Altered glutamate receptor - transporter expression and spontaneous seizures in rats exposed to methylazoxymethanol in utero. Epilepsia 48, 158–168. 10.1111/j.1528-1167.2006.00838.x 17241223

[B24] Holmes G. L. Gairsa J. L. Chevassus-Au-Louis N. Ben-Ari Y. (1998). Consequences of neonatal seizures in the rat: morphological and behavioral effects. Ann. Neurol. 44, 845–857. 10.1002/ana.410440602 9851428

[B25] Jöels M. (2009). Stress, the hippocampus, and epilepsy. Epilepsia 50, 586–597. 10.1111/j.1528-1167.2008.01902.x 19054412

[B26] Jellett A. P. Jenks K. Lucas M. Scott R. C. (2015). Standard dose valproic acid does not cause additional cognitive impact in a rodent model of intractable epilepsy. Epilepsy Res. 110, 88–94. 10.1016/j.eplepsyres.2014.11.005 25616460 PMC4397905

[B27] Lerner J. T. Salamon N. Hauptman J. S. Velasco T. R. Hemb M. Wu J. Y. (2009). Assessment and surgical outcomes for mild type I and severe type II cortical dysplasia: a critical review and the UCLA experience. Epilepsia 50, 1310–1335. 10.1111/j.1528-1167.2008.01998.x 19175385

[B28] Machin D. Campbell M. Fayers P. Pinol A. (1997). Sample Size Tables for Clinical Studies. Second Ed. Blackwell Sci. p, 24–25. 10.1002/(SICI)1097-0258(19990228)18:4<494::AID-SIM56>3.0.CO;2-T

[B29] Marchi N. Guiso G. Caccia S. Rizzi M. Gagliardi B. Noé F. (2006). Determinants of drug brain uptake in a rat model of seizure-associated malformations of cortical development. Neurobiol. Dis. 24 (3), 429–442. 10.1016/j.nbd.2006.07.019 17027274

[B30] Noè F. Cattalini A. Vila Verde D. Alessi C. Colciaghi F. Figini M. (2019). Epileptiform activity contralateral to unilateral hippocampal sclerosis does not cause the expression of brain damage markers. Epilepsia 60 (6), 1184–1199. 10.1111/epi.15611 31111475

[B31] Nobili P. Colciaghi F. Finardi A. Zambon S. Locatelli D. Battaglia G. S. (2015). Continuous neurodegeneration and death pathway activation in neurons and glia in an experimental model of severe chronic epilepsy. Neurobiol. Dis. 83, 54–66. 10.1016/j.nbd.2015.08.002 26264964

[B32] Patsalos P. N. Spencer E. P. Berry D. J. (2018). Therapeutic drug monitoring of antiepileptic drugs in epilepsy: a 2018 update. Ther. Drug Monit. 40, 526–548. 10.1097/FTD.0000000000000546 29957667

[B33] Paxinos G. Watson C. (1982). The Rat Brain in Stereotaxic Coordinates. 1st ed. (Sydney: Academic Press).

[B34] Pinel J. P. Rovner L. I. (1978a). Experimental epileptogenesis: kindling-induced epilepsy in rats. Exp. Neurol. 58, 190–202. 10.1016/0014-4886(78)90133-4 618743

[B35] Pinel J. P. Rovner L. I. (1978b). Electrode placement and kindling-induced experimental epilepsy. Exp. Neurol. 58, 335–346. 10.1016/0014-4886(78)90145-0 618751

[B36] Polli R. S. Malheiros J. M. Dos Santos R. Hamani C. Longo B. M. Tannús A. (2014). Changes in hippocampal volume are correlated with cell loss but not with seizure frequency in two chronic models of temporal lobe epilepsy. Front. Neurol. 5, 111. 10.3389/fneur.2014.00111 25071699 PMC4076745

[B37] Potschka H. (2012). Animal models of drug-resistant epilepsy. Epileptic Disord. 14 (3), 226–234. 10.1684/epd.2012.0532 22947487

[B38] Racine R. J. (1972). Modification of seizure activity by electrical stimulation: II. Motor seizure Electroencephalogr Clin. Neurophysiol. 32, 281–294. 10.1016/0013-4694(72)90177-0 4110397

[B39] Rossini L. Garbelli R. Gnatkovsky V. Didato G. Villani F. Spreafico R. (2017). Seizure activity per se does not induce tissue damage markers in human neocortical focal epilepsy. Ann. Neurol. 82 (3), 331–341. 10.1002/ana.25005 28749594

[B40] Serbanescu I. Cortez M. A. McKerlie C. Snead O. (2004). Refractory atypical absence seizures in rat: a two-hit model. Epilepsy Res. 62 (1), 53–63. 10.1016/j.eplepsyres.2004.08.003 15519132

[B41] Sharma S. Puttachary S. Thippeswamy A. Kanthasamy A. G. Thippeswamy T. (2018). Status epilepticus: behavioral and electroencephalography seizure correlates in kainate experimental models. Front. Neurol. 9, 7. 10.3389/fneur.2018.00007 29410648 PMC5787145

[B42] Sisodiya S. M. Heffernan J. Squier M. V. (1999). Over-expression of P-glycoprotein in malformations of cortical development. Neuroreport. 10 (16), 3437–3441. 10.1097/00001756-199911080-00032 10599858

[B43] Sisodiya S. M. Lin W. R. Squier M. V. Thom M. (2001). Multidrug-resistance protein 1 in focal cortical dysplasia. Lancet 357 (9249), 42–43. 10.1016/s0140-6736(00)03573-x 11197364

[B44] Sisodiya S. M. Lin W. R. Harding B. N. Squier M. V. Thom M. (2002). Drug resistance in epilepsy: expression of drug resistance proteins in common causes of refractory epilepsy. Brain 125 (Pt 1), 22–31. 10.1093/brain/awf002 11834590

[B45] Sloviter R. S. Zappone C. A. Harvey B. D. Frotscher M. (2006). Kainic acid-induced recurrent mossy fiber innervation of dentate gyrus inhibitory interneurons: possible anatomical substrate of granule cell hyper-inhibition in chronically epileptic rats. J. Comp. Neurol. 494, 944–960. 10.1002/cne.20850 16385488 PMC2597112

[B46] Sloviter R. S. Zappone C. A. Bumanglag A. V. Norwood B. A. Kudrimoti H. (2007). On the relevance of prolonged convulsive status epilepticus in animals to the etiology and neurobiology of human temporal lobe epilepsy. Epilepsia 48 (S8), 6–10. 10.1111/j.1528-1167.2007.01335.x 18329985

[B47] Smyth M. D. Barbaro N. M. Baraban S. C. (2002). Effects of antiepileptic drugs on induced epileptiform activity in a rat model of dysplasia. Epilepsy Res. 50, 251_/264. 10.1016/s0920-1211(02)00051-7 12200216

[B48] Sutula T. P. Hagen J. Pitkänen A. (2003). Do epileptic seizures damage the brain? Curr. Opin. Neurol. 16 (2), 189–195. 10.1097/01.wco.0000063770.15877.bc 12644748

[B49] Williams P. A. White A. M. Clark S. Ferraro D. J. Swiercz W. Staley K. J. (2009). Development of spontaneous recurrent seizures after kainate-induced status epilepticus. J.Neurosci. 29, 2103–2112. 10.1523/JNEUROSCI.0980-08.2009 19228963 PMC2897752

[B50] Zhang W. Huguenard J. R. Buckmaster P. S. (2012). Increased excitatory synaptic input to granule cells from hilar and CA3 regions in a rat model of temporal lobe epilepsy. J. Neurosci. 32, 1183–1196. 10.1523/JNEUROSCI.5342-11.2012 22279204 PMC3778651

